# Discharge Screening Predicts Persistent Parental Psychological Distress After Pediatric Critical Illness

**DOI:** 10.3390/children12101321

**Published:** 2025-10-02

**Authors:** Lynne M. Rosenberg, Gabrielle Silver, Keshia Small, Linda M. Gerber, Chani Traube

**Affiliations:** 1 Department of Poison & Drug Safety, Denver Health Medical Center, Denver, CO 80204, USA; 2 Department of Psychiatry, Weill Cornell Medical College, Cornell University, New York, NY 10065, USA; 3 Department of Pediatrics, Weill Cornell Medical College, Cornell University, New York, NY 10065, USA; 4 Department of Population Health Sciences, Weill Cornell Medical College, Cornell University, New York, NY 10065, USA

**Keywords:** pediatric, critical care, outcomes, family, parent, post-intensive care syndrome (PICS)

## Abstract

**Highlights:**

**What are the main findings?**

- **Parental psychological distress is common and persistent**: Many parents experienced anxiety and depression prior to PICU discharge, and 44% continued to screen positive for anxiety, depression, or post-traumatic stress symptoms more than 30 days after returning home.
- **Discharge screening has predictive value**: A positive screen for anxiety or depression at discharge independently predicted ongoing psychological symptoms at 30–60-day follow-up, suggesting a feasible and actionable strategy for identifying families at highest risk.

**What is the implication of the main finding?**

- **Opportunities for intervention**: Preventing long-term distress may be possible by supporting parents during their child’s ICU stay and using discharge screening to guide targeted follow-up psychosocial interventions.

**Abstract:**

**Background/Objectives**: Pediatric critical illness constitutes a highly stressful event not only for the critically ill child but also for the broader family unit. Recognizing and addressing these family-level effects is essential to optimizing outcomes for PICU survivors. **Methods**: This prospective observational cohort study examined anxiety and depression in parents of pediatric ICU patients prior to PICU discharge using the Hospital Anxiety and Depression (HADS) scale, and again after 30 days. At follow-up, parents were also screened for post-traumatic stress symptoms (PTSS) using the Impact of Events (IES) scale. Parent demographics and characteristics of the child’s hospitalization were collected. **Results**: 235 parents enrolled and completed a HADS; 126 parents (54%) subsequently completed a follow-up HADS and IES. 50% of parents screened positive for anxiety and/or depression prior to discharge; 44% of parents demonstrated anxiety, depression, and/or PTSS on follow-up screening. A positive HADS prior to discharge was the only independent predictor of persistent psychological symptoms. **Conclusions**: Parental psychological distress is both common and persistent following a child’s PICU admission. Screening at discharge is a feasible method to identify families at the highest risk for enduring anxiety, depression, and post-traumatic stress symptoms.

## 1. Introduction

Admission to the Pediatric Intensive Care Unit (PICU) constitutes a highly stressful event not only for the critically ill child but also for the broader family unit. Although advances in pediatric critical care have resulted in high survival rates, a substantial proportion of survivors experience persistent morbidities, including physical, neurocognitive, and psychosocial impairments [1,2,3,4]. Importantly, the effects of critical illness extend beyond the patient; parents and caregivers frequently endure significant emotional and psychological distress during and after their child’s PICU stay [5,6,7]. Emerging evidence suggests that this acute stress response may predispose parents to longer-term psychological morbidity, including symptoms of anxiety, depression, and post-traumatic stress disorder [8,9]. In turn, this may influence the child’s outcome, given the well-established link between parental and child mental health [10,11]. These risks are often compounded by social determinants of health, such as socioeconomic status, which can limit access to resources and supports, and by social isolation, which deprives families of essential networks of care [12,13]. Families’ coping strategies also vary widely, influencing resilience and recovery trajectories, while ongoing medical uncertainty surrounding prognosis or long-term outcomes may intensify distress [14]. Recognizing and addressing these family-level outcomes, and the contextual factors that shape them, is therefore essential to a comprehensive understanding of the sequelae of pediatric critical illness.

To optimize post-discharge support for critically ill children and their families, it is essential to identify those parents at highest risk for psychological morbidity. Early identification of vulnerable families would enable targeted follow-up interventions and the development of evidence-based support strategies. However, a substantial knowledge gap remains regarding which patient- or family-level characteristics confer the greatest risk for adverse psychological outcomes [15]. The primary objective of this study was to evaluate symptoms of anxiety and depression in parents of PICU survivors at the time of hospital discharge and again 30–60 days following the PICU stay. The secondary objectives were to (i) describe the associations between characteristics of the hospital stay and parental symptoms prior to discharge and (ii) assess the relationship between anxiety and/or depression at discharge and the presence of anxiety, discharge, or post-traumatic stress symptoms at 30–60 days. We hypothesized that a significant proportion of parents would already demonstrate symptoms consistent with anxiety and depression at the point of discharge, and that this subgroup would be at the highest risk for persistent psychological morbidity over time.

## 2. Materials and Methods

Study Design

This prospective, observational cohort study took place in an urban academic tertiary care hospital with a dedicated Pediatric Intensive Care Unit (PICU). The study was conducted in accordance with the Declaration of Helsinki and approved by the Institutional Review Board of the Weill Cornell Medical College (IRB# 1711018718). Informed consent was obtained from all subjects involved in the study.

Participants

Eligible participants included the primary caregiver (parent or legal guardian, hereafter referred to as “parent”) of any child admitted to the PICU for greater than 48 h. Parents with limited written English proficiency were excluded from the study.

Procedures

Parents were approached within 72 h of their child’s anticipated discharge and invited to participate. After written informed consent was obtained, parents completed the Hospital Anxiety and Depression Scale (HADS) at the time of discharge [16]. At 30–60 days following discharge, parents were re-contacted to complete the HADS again, as well as the Impact of Event Scale—Revised (IES-R) [17].

Instruments

*Hospital Anxiety and Depression Scale* (HADS) [16]: A validated 14-item instrument assessing symptoms of anxiety and depression; requires approximately five minutes to complete.

*Impact of Event Scale—Revised* (IES-R) [17]: A validated 22-item questionnaire designed to evaluate post-traumatic stress symptoms in adults.

Scoring and Follow-Up

Study personnel scored all questionnaires. A pediatrician, serving as a study investigator, reviewed all concerning results. Participants with clinically significant scores on follow-up assessments were referred for further psychological evaluations. To ensure continuity of care, the child’s primary outpatient pediatrician was also notified as a secondary point of contact for families requiring further evaluation.

Data Collection

Demographic data, including education level, marital status, religiosity, and history of prior mental health diagnoses, were obtained by parent self-report. Clinical data were abstracted from the electronic medical record (EMR), including patient demographics, admitting diagnosis, and delirium status (yes/no, as determined by a score of ≥9 on the *Cornell Assessment of Pediatric Delirium*, CAPD, during the PICU stay) [18]. Severity of illness was defined by the Pediatric Index of Mortality III [19].

Statistical Methods

Parent and child demographic and clinical characteristics were summarized using descriptive statistics. Categorical variables were reported as frequencies and percentages, while continuous variables were summarized using means with standard deviations, or medians with ranges, as appropriate.

Univariate logistic regression models were used to examine associations between predictor variables and dichotomized outcomes of anxiety, depression, and post-traumatic stress disorder (PTSD) (coded as present/absent) at either time-point. Operational definition of a positive HADS screen was a score of ≥8, and PTSD screen was a score of ≥24. Predictor variables of interest included parent role (mother/father), race, level of education, self-described religiosity (dichotomized as ‘religious’, including any response indicating religiosity, versus ‘not religious’), and relationship status, as well as parental history of anxiety, trauma, and/or depression prior to index admission (yes/no). In addition, patient variables of interest included the child’s age, diagnosis, severity of illness, and presence of delirium (yes/no).

Multivariable logistic regression models were then constructed for outcomes of interest, incorporating predictor variables determined a priori, as well as all variables that reached a significance of 0.1 in univariate analyses. A model was constructed to evaluate the relationship between parental anxiety and/or depression at discharge and the development of parental anxiety/depression/PTSS at >30 days. All tests were two-sided, with statistical significance defined as *p* < 0.05. Exact 95% confidence intervals were reported for all effect estimates to assess precision. Analyses were performed using SAS version 9.3 (SAS Institute Inc., Cary, NC, USA).

## 3. Results

Study Cohort

Two hundred and thirty-five parents enrolled in the study. 77% (*n* = 180) of those who consented were mothers. Forty-seven percent of parents were White, 20% were Black, and 12% were Asian. One quarter of the cohort (25.5%) described themselves as Hispanic/Latino. Most participants were married (68%), and 72% had more than one child. Sixty-two percent (*n* = 145) of the cohort described themselves as religious. Most parents were highly educated; 55% had an undergraduate degree. A few of the parents had prior diagnoses of anxiety (11%), depression (7%), and/or PTSD (4%).

Upon discharge, all 235 parents completed a HADS screen. Overall, 49.8% of parents (*n* = 117) screened positive on the HADS. This included 22% (*n* = 52) of parents who screened positive for depression and 46% for anxiety. Parental characteristics associated with a positive screen included a prior mental health diagnosis, higher education, and a marital relationship characterized as separated or divorced. Parents who described themselves as Black had lower rates of anxiety and depression prior to discharge. There was no association between anxiety/depression and religious beliefs. Although it did not reach statistical significance, there were notably higher rates of anxiety/depression in parents of older children (56% in ≥5 years vs. 45% in <5 years, *p* = 0.1) and in parents of children who experienced delirium during the hospitalization (66% vs. 48%, *p* = 0.07). There was no relationship between parental anxiety/depression and patient characteristics such as admission diagnosis or severity of illness (Table 1).

One hundred and twenty-six parents (53.6%) completed a follow-up screen at >30 days after discharge home. Categorical demographic and clinical variables were compared between the initial cohort and the follow-up cohort using chi-square tests of independence. Across most variables, there were no statistically significant differences between groups, indicating that the follow-up sample was broadly representative of the original cohort. The only significant difference was observed in parental education level: a higher proportion of parents with an undergraduate degree participated in the follow-up assessment compared with the initial cohort (67% vs. 55%, *p* = 0.025).

Overall, 44% of parents (*n* = 56) screened positive on at least one follow-up assessment. More than 30 days after PICU discharge, parents had high rates of clinically significant symptoms: 32% with anxiety, 27% with depression, and 13% with PTSS. Similar to results prior to discharge, parents with a prior mental health diagnosis were more likely to screen positive for anxiety, depression, and/or PTSS (75% vs. 40%, *p* = 0.008). In univariate analysis, mothers had higher rates of positive screens than fathers (51% vs. 28%, *p* = 0.035). However, higher education, marital status, race, and the child’s age were no longer associated with a positive psychological screen. Rates of persistent psychological symptoms tended to be higher in parents who witnessed delirium in their child, although this association narrowly missed statistical significance (10/15, 67%, vs. 46/111, 41%, *p* = 0.06). Notably, parents with a positive screen for anxiety and/or depression at discharge had much higher rates of anxiety, depression, and/or PTSS at >30 days (67% vs. 22% amongst those without a prior positive screen, *p* < 0.001). See Table 2 for a description of the parents who enrolled in the study and completed follow-up.

**Figure 1:**
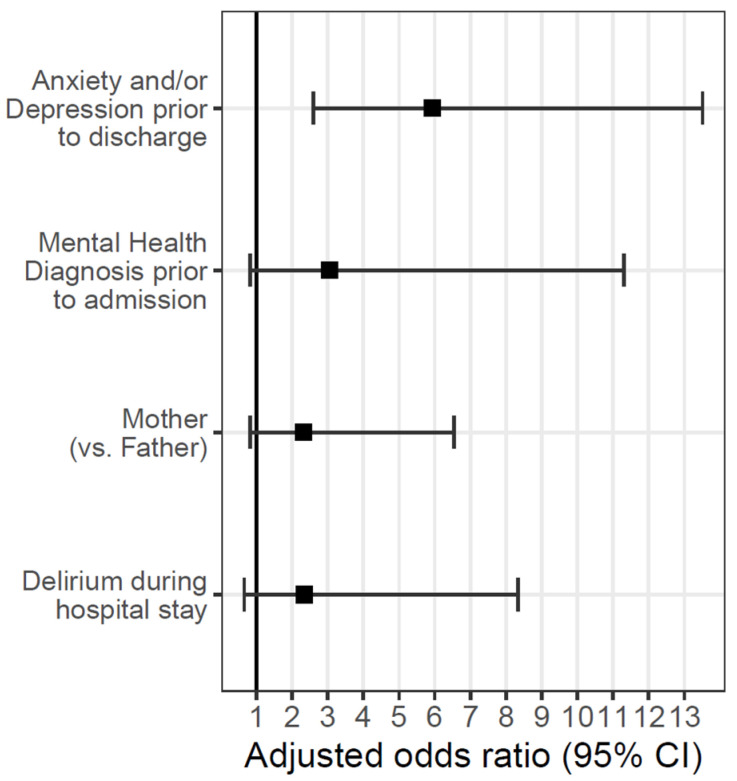
Forest plot demonstrating the relationship between parent and patient characteristics and adjusted odds of screening positive for anxiety, depression, or post-traumatic stress symptoms > 30 days after discharge from the PICU.

The multivariable model, including all variables that reached a *p*-value of ≤0.1, demonstrated that only a positive screen for anxiety or depression at discharge independently predicted anxiety, depression, or post-traumatic stress symptoms at >30-day follow-up (Figure 1).

## 4. Discussion

This study adds important new evidence to the growing literature on family outcomes after pediatric critical illness. We found that many parents of PICU patients experienced significant symptoms of anxiety and depression prior to discharge, and more than 40% of parents endorsed clinically meaningful anxiety, depression, or post-traumatic stress symptoms more than 30 days after returning home. These findings confirm that the psychological impact of a child’s critical illness persists well beyond hospitalization. As there is a strong and evidence-based literature describing the impact of anxious/depressed parents on children’s well-being, this parental distress is likely to negatively impact the patient and family during the crucial transition from hospital to home [10,11].

Most notably, we identified that screening for anxiety and depression at the time of PICU discharge independently predicted the presence of anxiety, depression, or post-traumatic stress symptoms at >30-day follow-up. This is an actionable finding. While prior studies have consistently documented high rates of parental psychological distress following a child’s critical illness, few have examined whether symptoms identified at discharge can serve as a reliable predictor of ongoing morbidity [5,6,7,9,20]. Instead, prior work has largely focused on demographic risk factors (such as socioeconomic status), social factors (such as isolation and/or marital cohesion), or illness-related variables (such as severity of illness and/or medical uncertainty) [5,6,7,12,13,14,21]. These factors, while important, are not easily modifiable and may be less actionable in the clinical setting. By contrast, our results suggest that a brief, parent-reported screen at discharge is both feasible and clinically meaningful, offering a new tool for identifying families most at risk for enduring symptoms.

Implications for clinical care

This finding has direct clinical relevance. Discharge is already a structured care transition, and adding a brief standardized screen could be integrated into routine workflows with minimal burden [22]. Parents who screen positive could be referred for targeted psychosocial interventions, including evidence-based therapies aimed at reducing anxiety, depression, or post-traumatic stress symptoms; structured mental health support such as counseling, peer support groups, or access to community resources; and longitudinal follow-up with pediatricians, psychologists, or social workers to monitor ongoing needs and facilitate early intervention if difficulties persist [23,24]. Such an approach would align with family-centered models of PICU care and with broader recognition of Post-Intensive Care Syndrome in pediatrics (PICS-p), in which family mental health is an essential domain of recovery [1,8,25]. By identifying vulnerable families early, pediatric critical care teams may help mitigate long-term psychological sequelae in parents, which in turn could have downstream benefits for children [21].

Opportunities for prevention during the ICU stay

In addition to screening at discharge, our findings highlight the need to address parental distress during the ICU stay itself. Psychological symptoms often develop while the child is critically ill, fueled by uncertainty, fear, disrupted family routines, and the overwhelming ICU environment [26,27,28]. Interventions such as structured family support, improved communication, parent participation in care, and early referral to social work or psychology services may help buffer stress before it becomes entrenched [27,28,29]. Identifying vulnerable parents and preventing or reducing distress during the admission may lower the number of parents who screen positive at discharge, thereby reducing long-term morbidity [27]. Future studies should examine whether combining proactive support during hospitalization with targeted screening at discharge offers an important strategy for improving parent and family outcomes.

Limitations and future directions

An important limitation of our cohort is that parents who completed follow-up were disproportionately highly educated. This may not be representative of the wider population of families with critically ill children, and could introduce selection bias and/or concerns about external validity. This differential participation raises uncertainty about the generalizability of our findings. It is possible that those who responded were the most distressed, and thus more motivated to engage with follow-up efforts and with PICU staff. Conversely, it is also plausible that respondents were those least distressed, with greater time, resources, and fewer inhibitions about re-engaging with the PICU environment. Both scenarios suggest the potential for nonresponse bias, a challenge well recognized in psychosocial and survey-based research [30]. We were not able to include parents with limited English proficiency in this study. As a result, the prevalence of parental psychological symptoms observed in this study may under- or over-estimate the true burden across all PICU parents. Future multi-center studies should include broader and more diverse samples, as well as strategies to decrease attrition bias and improve follow-up participation. This is important to ensure that findings are more representative and applicable across varied sociodemographic groups.

Several other limitations must be acknowledged. Our findings are based on a single cohort and may not be generalizable to diverse healthcare settings, cultural backgrounds, and family structures. While we used validated screening tools, they are less robust and do not replace formal diagnostic assessments. We did not have pre-admission symptom data and relied on parents’ self-reports of preexisting mental health diagnoses. We did not collect potentially influential variables related to socioeconomic status, coping strategies, and/or concurrent stressors that may have affected screening results. The follow-up period was limited to 30–60 days; longer-term trajectories remain unknown. Furthermore, attrition may have biased results toward families with greater stability and resources. Future work should confirm these findings in larger, multi-center cohorts, extend follow-up to capture long-term recovery, and evaluate the effectiveness of preventive interventions initiated during the ICU stay.

## 5. Conclusions

In summary, we demonstrate that parental psychological distress is both common and persistent following a child’s PICU admission, and that screening at discharge is a feasible method to identify families at highest risk for enduring anxiety, depression, and post-traumatic stress symptoms. These findings contrast with prior studies that have emphasized less modifiable demographic or illness-related risk factors, underscoring the unique actionability of discharge screening. Moreover, preventing distress may be possible by supporting families during the ICU stay itself, suggesting a dual strategy of prevention and early identification. Together, these insights open new avenues for family-centered intervention at critical points in care, with the potential to improve outcomes for both parents and their children.

## Figures and Tables

**Table 1:** Association of parent/patient characteristics and parental anxiety/depression prior to discharge (*n* = 235).

Variable	Category	Anxiety and/or Depression (*n* = 117)	Negative Screen (*n* = 118)	*p*-Value
Parent role *(n = 233)*	Mother	94 (52.2%)	86 (47.8%)	0.2
	Father	23 (43.4%)	30 (56.6%)	
Education	No undergraduate degree	41 (38.3%)	66 (61.7%)	0.001
	Undergraduate degree or higher	76 (59.4%)	52 (40.6%)	
Race	White	65 (59.1%)	45 (40.9%)	0.003
	Asian	17 (60.7%)	11 (39.3%)	
	Black	14 (29.2%)	34 (70.8%)	
	Other/Unknown	21 (42.9%)	28 (57.1%)	
Ethnicity	Hispanic/Latino	26 (43.3%)	34 (56.7%)	0.25
	Not Hispanic/Latino	91 (52%)	84 (48%)	
Relationship Status *(n = 233)*	Single	18 (31.6%)	39 (68.4%)	<0.001
	Married	85 (53.1%)	75 (46.9%)	
	Separated/Divorced	13 (81.2%)	3 (18.8%)	
Religious status (self-reported) *(n = 233)*	Yes	71 (49%)	74 (51%)	0.75
	No	45 (51%)	43 (49%)	
Prior Mental Health Diagnosis	Yes	23 (67.6%)	11 (32.4%)	0.024
	No	94 (46.8%)	107 (53.2%)	
**Patient-Related Variables**				
Child’s Age	<5 years	58 (45%)	71 (55%)	0.1
	≥5 years	59 (55.7%)	47 (44.3%)	
Admission Diagnosis Category	Respiratory insufficiency/failure	55 (49.5%)	56 (50.5%)	0.46
	Neurologic disorder	39 (52.7%)	35 (47.3%)	
	Cardiac disease	10 (35.7%)	18 (64.3%)	
	Other	13 (59.1%	9 (40.9%)	
Severity of Illness	Pediatric Index of Mortality III) (mean +/− SD)	2% (+/−7%)	1% (+/−3%)	0.33
Delirium	Yes	19 (65.5%)	10 (34.5%)	0.07
	No	98 (47.6%)	108 (52.4%)	

**Table 2:** Association of parent/patient characteristics and parental anxiety/depression at >30 days follow-up (*n* = 126).

Variable	Category	Anxiety and/or Depression (*n* = 56)	Negative Screen (*n* = 70)	*p*-Value
Parent role	Mother	48 (50.5%)	47 (49.5%)	0.035
	Father	8 (26.7%)	22 (73.3%)	
Education	No undergraduate degree	15 (35.7%)	27 (64.3%)	0.16
	Undergraduate degree or higher	41 (48.8%)	43 (51.2%)	
Race	White	31 (43.1%)	41 (56.9%)	0.43
	Asian	3 (27.3%)	8 (72.7%)	
	Black	11 (45.8%)	13 (54.2%)	
	Other/Unknown	11 (57.9%)	8 (42.1%)	
Ethnicity	Hispanic/Latino	14 (46.7%)	16 (53.3%)	0.78
	Not Hispanic/Latino	42 (43.8%)	54 (56.2%)	
Relationship Status	Single	13 (54.2%)	11 (45.8%)	0.54
	Married	40 (42.1%)	55 (57.9%)	
	Separated/Divorced	3 (42.9%)	4 (57.1%)	
Religious status (self-reported)	Yes			
	No			
Prior Mental Health Diagnosis	Yes	12 (75%)	4 (25%)	0.008
	No	44 (40%)	66 (60%)	
**Patient-Related Variables**				
Child’s Age	<5 years	31 (40.8%)	45 (59.2%)	0.31
	≥5 years	25 (50%)	25 (50%)	
Admission Diagnosis Category	Respiratory insufficiency/failure	24 (43.6%)	31 (56.4%)	0.52
	Neurologic disorder	19 (45.2%)	23 (54.8%)	
	Cardiac disease	8 (44.4%)	10 (55.6%)	
	Other	5 (45.5%)	6 (54.5%)	
Severity of Illness	Pediatric Index of Mortality III) (mean +/−SD)	3% (+/−1%)	2% (+/−5%)	0.73
Delirium	Yes	10 (66.7%)	5 (33.3%)	0.06
	No	46 (41.4%)	65 (58.6%)	
**Positive Screen for Anxiety/Depression Prior to Discharge**	Yes	42 (66.7%)	21 (33.3%)	<0.001
	No	14 (22.2%)	49 (77.8%)	

## Data Availability

The data that support the findings of this study are available from the corresponding author upon reasonable request.
